# France-Wide Monitoring of 1,4-Dioxane in Raw and Treated Water: Occurrence and Exposure Via Drinking Water Consumption

**DOI:** 10.1007/s00244-024-01078-6

**Published:** 2024-07-31

**Authors:** Cristina Bach, Virginie Boiteux, Xavier Dauchy

**Affiliations:** Nancy Laboratory for Hydrology, Water Chemistry Unit, ANSES, 40 rue Lionnois, 54000 Nancy, France

## Abstract

In recent years, 1,4-dioxane has emerged as a pollutant of increasing concern following widespread detection in the aquatic environment of several countries. This persistent contaminant with specific physical and chemical properties can be rapidly dispersed and transported to river banks, groundwater and drinking water. Given the limited data on its occurrence in France, it was considered necessary to assess the potential exposure of the French population to this compound in drinking water. An analytical method based on solid-phase extraction (SPE) combined with gas chromatography tandem mass spectrometry (GC–MS/MS) was developed and validated during this study with a limit of quantification (LOQ) of 0.15 µg/L. Recoveries in natural water matrices ranged from 113 to 117% with a relative bias not exceeding 17%. This method was used for a nationwide campaign at almost 300 sites, evenly distributed over 101 French *départements* (administrative units), including some that were overseas. Of the 587 samples analysed, only 8% had a concentration that was greater than or equal to the LOQ. 1,4-Dioxane was detected mainly (63%) in raw and treated water from sites associated with historical industrial practices related to the use of chlorinated solvents. Concentrations of 1,4-dioxane ranging from 0.19 to 2.85 µg/L were observed in the raw water and from 0.18 to 2.46 µg/L in the treated water. Drinking water treatment plants using ozonation, granular activated carbon and chlorination have limited effectiveness in the removal of 1,4-dioxane. The results of this study are the first step towards bridging the knowledge gap in the occurrence of 1,4-dioxane in France.

**Supplementary Information:**

The online version contains supplementary material available at 10.1007/s00244-024-01078-6.

1,4-Dioxane is a synthetic compound considered to be one of the major emerging pollutants in the environment. This compound was historically used as a stabilizer for chlorinated solvents in industrial processes such as 1,1,1-trichloroethane (TCA) (Adamson et al. 2014; Godri Pollitt et al. 2019; Karges et al. 2018; Mohr et al. 2010; USEPA 2013). The use of TCA was reduced by the Montreal Protocol in 1995 due to its ozone-depleting potential (Arulazhagan et al. 2013; ITRC 2021; USEPA 2014) resulting in a significant reduction in the production of 1,4-dioxane. 1,4-Dioxane is still currently used directly or as an additive in the rubber and plastics industries, in automotive fluids and inks, paints and coatings among others industries.

1,4-Dioxane also occurs as a by-product in pesticides, pharmaceutical formulation and food contact packaging processes, and in some forms of acetate and ethoxylated surfactant production (ITRC 2021; Mohr et al. 2010; USEPA 2014).

It is considered to be a persistent and mobile organic compound in the aquatic environment due to its intrinsic chemical properties (see Table S1). Once released into the environment, 1,4-dioxane can be rapidly dispersed and transported through river banks and groundwater into drinking water resources and remote aquatic systems. As a result, it can end up in drinking water, potentially posing a threat to human health (Hale et al. 2020; Kim et al. 2023; Neuwald et al. 2022).

The US Environmental Protection Agency (US EPA) and the International Agency for Research on Cancer (IARC) have classified 1,4-dioxane as “*probably to be carcinogenic to humans*” by all routes of exposure (IARC 1976). Contaminated drinking water is one of consumers’ main routes of exposure to 1,4-dioxane (Doherty et al. 2023; Godri Pollitt et al. 2019; USEPA 2014). Indeed, this chemical is completely soluble in water and does not volatilize (McElroy et al. 2019).

Drinking water guideline values for 1,4-dioxane vary widely from country to country. Canada, Japan, the Republic of Korea and the World Health Organization have set a guideline value of 50 µg/L (Godri Pollitt et al. 2019). The US Environmental Protection Agency (US EPA) set an Integrated Risk Information System (IRIS) drinking water screening concentration of 0.35 µg/L (USEPA 2014) and a tap water regional screening level (RSL) of 0.46 µg/L (USEPA 2013). Both levels are calculated based on an acceptable cancer risk of one in 1 million. Risk-based concentrations vary because of differences in assumed exposure pathways and exposure frequency (Broughton et al. 2019). The German Environmental Agency has been more restrictive by suggesting a guideline value of 0.1 µg/L (Karges et al. 2018; McElroy et al. 2019). (ECHA 2021a, 2021b). In Europe, 1,4-dioxane has been included on the candidate list as a *substance of very high concern* (SVCH) in the article 59(10) of the REACH regulation and the next step would be to incorporate it on the authorization list (Annex XIV of the REACH regulation) (EU 2006).

The analysis of 1,4-dioxane in water is challenging, leading to analytical methods with high quantification limits and low recovery rates (Hayes et al. 2022; USEPA 2014). Thus, the US and German reference levels (0.35 and 0.1 µg/L) in drinking water cannot be easily achieved (Adamson et al. 2021).

The instrumental analysis of 1,4-dioxane in water is essentially carried out by gas chromatography coupled to mass spectrometry (GC–MS). For aqueous environmental samples, various extraction techniques such as solid-phase extraction (SPE) and purge and trap (P&T) have been reported over the years, as reviewed by Sun et al. (2016) and McElroy et al. (2019). In Table 2S, a non-comprehensive summary of extraction methods for 1,4-dioxane was presented.

The occurrence of 1,4-dioxane in the aquatic environment has been investigated in only a few countries. In the USA, the third unregulated contaminant monitoring rule programme (UCMR 3) collected data on several contaminants from US public drinking water supplies (USEPA 2020). Covering three years, the data set showed that 1,4-dioxane was present in 21% of the public water systems sampled (4,864 public water systems). Compared with other contaminants investigated in UCMR 3, the detection frequency of 1,4-dioxane was relatively high, resulting in 6.9% of observed concentrations exceeding the US EPA reference concentration of 0.35 µg/L (Adamson et al. 2017, 2021). More recently, the systematic occurrence of 85 volatile compounds was investigated in aquifers feeding US public water supplies. With a limit of detection of 0.35 µg/L for 1,4-dioxane, its frequency of detection was 0.5% of the groundwater sampled (nine wells) in the California coastal basins (Bexfield et al. 2022). In Germany, groundwater contaminated with 1,4-dioxane has also been reported at sites where chlorinated solvents were previously used or produced (De Boer et al. 2022; Karges et al. 2018). The maximum observed concentration was 152 µg/L at one site. In 2017–2018, the same research group conducted a study of 1,4-dioxane in surface water and associated treated water after the implementation of mitigation measures at several sites. A decrease in 1,4-dioxane was observed, with concentrations below 10 µg/L in surface water and 1.68 µg/L in drinking water (Karges et al. 2022). In Spain, a survey conducted in 2015 showed 1,4-dioxane concentrations between 5.7 and 11.6 µg/L in groundwater from the Llobregat River. The concentrations of 1,4-dioxane observed were far above the LOQ of the analytical method at 50 ng/L in surface water (Carrera et al. 2017, 2019). In China, from May 2018 to April 2019, 15 sampling sites were investigated along a river that supplies the city of Shanghai City. Surface water samples showed 100% of detection of 1,4-dioxane with a maximum concentration of 8.3 µg/L (Wang et al. 2022). In France, 1,4-dioxane surface water monitoring data are collected via the Naïades information portal (Naïades 2023). However, depending on the laboratory in charge of the determination, LOQs ranged from 0.5 to 15 µg/L. Consequently, 1,4-dioxane was quantified in only 0.3% of the surface waters analysed. Concentrations over 20 µg/L were recorded in a river likely tainted by discharge from a known pharmaceutical plant. Given the environmental problems and the growing concern for human health posed by 1,4-dioxane and the lack of data on its occurrence in France, it is clearly necessary to assess the potential exposure of the French population to this compound in drinking water. The objectives of this study were therefore: (i) to develop and validate an analytical method for 1,4-dioxane in natural water matrices with a LOQ of 0.15 µg/L (below the US EPA guideline of 0.35 µg/L) and (ii) to carry out a sampling campaign to determine the presence of 1,4-dioxane in public water supplies throughout France.

## Material and Methods

### Chemicals

1,4-dioxane and 1,4-dioxane-d_8_ were purchased as solutions in methanol (1,000 to 10,000 µg/mL) from Dr Ehrenstorfer GmbH (Augsburg, Germany) via LGC Standards (Molsheim, France). Methanol ULC-MS (MeOH) and ethyl acetate LC–MS (EtAc) were purchased from Biosolve (Dieuze, France). Dichloromethane Pestinorm® (DCM) was purchased from VWR International (Rosny-sous-Bois, France). Ultrapure pure water was produced by a Millipore Milli-Q® Integral 10 water purification system (Milford, MA, USA).

Intermediate stock solutions of 1,4-dioxane at concentrations of 10 and 100 mg/L were prepared in MeOH using volumetric flasks and then transferred to 2 mL amber glass vials and stored at − 18 °C to limit evaporation.

Calibration points from 5 to 500 µg/L were prepared in a solvent mixture (v/v) of 80% DCM and 20% EtAc. An internal standard calibration was carried out using 1,4-dioxane-d_8_ at 100 µg/L in water samples.

### Sampling Strategy

This study was conducted on almost 300 sites spread evenly across 101 French *départements* including some that were overseas, from which raw and treated water was systematically collected.

For each *département,* three sample locations were investigated:

1. The water catchment producing the greatest flow of treated water (204 water samples),
2. a randomly selected drinking water source (190 water samples),
3. additional samples potentially contaminated by 1,4-dioxane industrial activities (193 water samples).

In all, 300 raw water samples and 287 treated water samples were analysed in this sampling campaign from October 2020 to February 2022. In line with the distribution of water catchment areas in France, about 2/3 of the raw water samples were taken from groundwater resources, and 1/3 from surface water resources. Since the sampling strategy encompassed, the resources providing the highest flow of each French *département* samples are representative of about 20% of the water distributed to the French population.

It is worth noting that a few drinking water treatment plants may be supplied by more than one raw water source, such as in the case of a wellfield. Due to the sampling strategy, only one raw water sample was collected from each site, thus providing limited information on the raw water quality supplying this type of treatment plant.

**Fig. 1:**
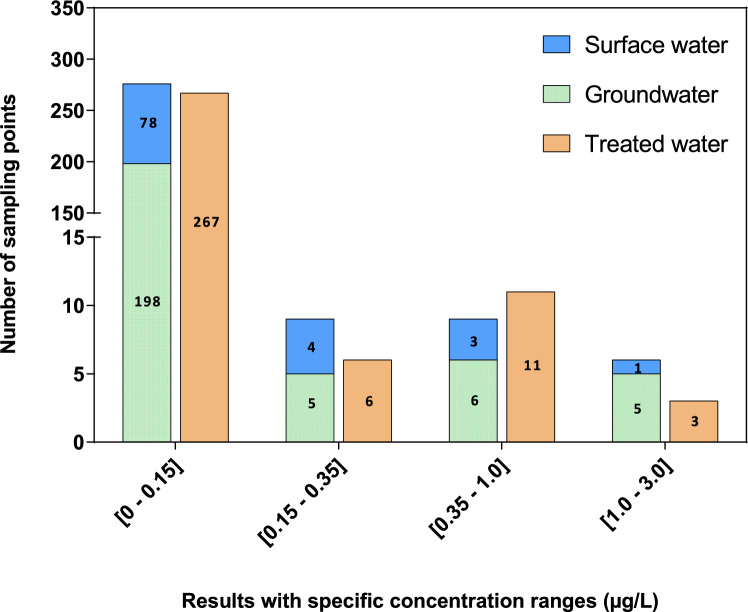
1,4-Dioxane results with specific concentration ranges for the 587 water samples (surface water, groundwater and treated water) analysed during the sampling campaign

All the water samples were collected in amber glass bottles (1 L). Sodium thiosulphate was added to treated water in order to quench free chlorine. The water temperature of all the samples was measured in the field. Total and free chlorine were determined only in treated water samples (data not shown). Samples were shipped with cold packs and dispatched to the laboratory within 24–48 h. Samples were stored in the dark at 4 °C until analysis. A stability study conducted at 4 °C in surface water, groundwater and treated water with sodium thiosulphate has shown that 1,4-dioxane is stable over a period of 3 months (see Figure 1S).

### Analytical Method

SPE was performed on a Gilson’s GX-274 ASPEC™ instrument (Middleton, WI, USA) using Supelclean™ coconut charcoal cartridges (2 g adsorbent, 6 mL) purchased from Merck (Bellefonte, PA, USA). The SPE extraction was performed according to EPA Method 522 (USEPA 2008) with some modifications described below. The cartridges were conditioned successively with 5 mL of DCM, 5 mL of MeOH and 12 mL of ultrapure water. Five hundred mL of sample was spiked with 5 µL of a solution of 1,4-dioxane-d_8_ at 100 mg/L in MeOH and loaded onto the SPE cartridge at a flow rate of 7 mL/min. After this step, the cartridges were dried with a stream of nitrogen for 30 min. After elution with 9 mL of DCM (2 × 4.5 mL), more than 50% of the extracted volume was naturally evaporated under a hood overnight. The DCM extracts were then frozen at − 18 °C. Once the residual water was frozen, the DCM was removed from residual water with a Pasteur pipette transferred to volumetric tubes and made up to 5 mL with a solvent mixture (v/v) of 80% DCM and 20% EtAc. The extracts were immediately analysed or stored at − 18 °C.

Analyses were performed using a 7890B gas chromatograph (GC) coupled to a 7000D triple quadrupole mass spectrometer (MS) from Agilent Technologies (Santa Clara, CA, USA) equipped with a Gerstel MPS 2 Autosampler (Mülheim an der Ruhr, Germany). A Rxi-624MS column (30 m × 0.25 mm; 1.4 µm) from Restek (Bellefonte, PA, USA) was used for the chromatographic separation with the following oven programme: 35 °C (hold 2 min) to 90 °C at 10 °C/min, then to 270 °C at 40 °C/min and finally 270 °C hold 1 min. Three µL of the sample extract was injected at 200 °C for 2.5 min in splitless mode. The helium flow rate was set to 1.4 mL/min. The MS was operated in electron impact (EI) ionization mode (70 eV) using multiple reaction acquisition monitoring (MRM). Ion source and transfer line temperatures were set at 200 °C and 280 °C, respectively. Nitrogen was used as the collision gas at a flow rate of 1.5 mL/min, while helium was used as the quench gas at a flow rate of 2.25 mL/min. The specific MS/MS transitions are given in Table 1. The chromatograms of 1,4-dioxane with retention time and MRM transition peaks are shown in Fig. 3S.

**Table 1:** Analytes with their CAS registry number, molecular weight (MW), retention time (RT) and MRM transitions

	CAS	MW (Da)	RT (min)	Quantification transition (Q)	Confirmation transition (q1)	Confirmation transition (q2)
1,4-dioxane	123-91-1	88	8.05	88 > 88	88 > 58	88 > 57
1,4-dioxane-d_8_	17647-74-4	96	7.95	96 > 96	–	–

### Performance of the Analytical Method

The SPE-GC/MSMS method with a LOQ of 0.15 µg/L was validated during our study by applying the requirements of French standard NF T 90-210 (NF 2018) using natural representative matrices (surface water, groundwater and tap water) under intermediate precision to demonstrate the reliability of the analytical data. The method was accredited by the French Accreditation Committee (COFRAC) in 2020.

The range of the calibration curve was studied with five calibration points ranging from 15 to 500 µg/L in a solvent mixture (v/v) of 80% DCM and 20% EtAc for 1,4-dioxane. The second order nonlinear internal standard calibration function was performed six times (on different days) from standard solutions freshly prepared each day. The correlation coefficients (*r*^2^) obtained were ≥ 0.98. The back-calculated concentrations between the experimental and the nominal values must be within ± 15% for all calibration points, and within ± 20% for the calibration point corresponding to the LOQ at 0.15 µg/L. The results were acceptable and are presented in Fig. S2.

The limit of detection (LOD) was not considered in this study due to the low limit of quantification (LOQ) of 0.15 µg/L. The LOQ was defined as the lowest concentration of the analyte that can be determined with acceptable precision according to the French standard NF T 90-210 (NF 2018). The LOQ for 1,4-dioxane was validated under intermediate precision conditions in natural matrices. Six water samples (two groundwater, two surface water and two drinking water samples) were spiked at the pre-established LOQ. Inter-day precision was performed by analysing six series of duplicate extractions on six different days. To ensure the accuracy (trueness and precision), a maximum allowed tolerance (MAT) between the theoretical and the experimental values at the LOQ must not exceed ± 60%. The MAT was fixed as requested by the NF T90-210 standard, and its calculation was described in detail by Lardy-Fontan et al. (2018) and Mirmont et al. (2023). The LOQ of 0.15 µg/L for 1,4-dioxane was validated.

Following the same procedure, the accuracy of the method was also evaluated for two intermediate concentrations of the calibration range (0.75 and 4 µg/L). In this case, the MAT did not exceed ± 40% for these two concentration levels.

The relative recovery study was carried out by spiking groundwater, surface water and drinking water at 0.15 µg/L, 0.75 µg/L and 4 µg/L in duplicates. Mean recoveries were calculated using the three matrices for each spiking concentration. As shown in Table 3S, the mean recoveries for 1,4-dioxane ranged from 117 to 114% for the three 1,4-dioxane concentrations studied. In addition, during the sampling campaign, several water samples of each batch were spiked with 1,4-dioxane at 1 µg/L in order to evaluate recoveries in the experimental conditions with different real matrix samples. Recovery results with their standard deviation are listed in Table 4S. It is worth noting that average recoveries are calculated with 92 different matrix samples (treated water, surface and groundwater) and over a period of 1 year and 4 months (reproducibility conditions).These experimental recoveries were within the limits (70% and 120%) set by ISO 21253-2:2019 (ISO 2019).

The relative uncertainty (*U)* was calculated in order to compare the measured results. The uncertainty was extended by a coverage factor (*k*) of 2 (95% confidence level). The measured uncertainty was 47% for the LOQ and 30% for the two intermediate concentrations in the calibration range.

### Quality Control

Procedural blanks were prepared using ultrapure water in 1 L amber collection bottles and stored at 4 °C. For each sample batch, 500 mL of this water was analysed using the SPE procedure described above. These procedural blanks were used to check for possible contamination from the sample containers and the entire analytical procedure.

Within-run and intra-sample controls were systematically performed for each sample batch. The within-run controls consisted of calibration check standards inserted throughout the sample batch at 50 µg/L DCM/EtAc (80:20) for 1,4-dioxane. To validate the batch, the bias between the experimental and the theoretical concentration must be ≤ 20%. Intra-sample controls consisted of spiking some of the collected water samples at 0.15 µg/L (LOQ) and at 1 µg/L with 1,4-dioxane in order to check the trueness of the method. These intra-sample controls were considered valid if their recoveries were between 70 and 120% according to ISO 21253-2:2019 (ISO 2019).

Identification of 1,4-dioxane was confirmed according to the requirements of ISO 21253-1:2019: (i) the relative retention time of the target compounds must match that of the calibration points with a tolerance of ± 2.5%, and (ii) the abundance ratio (based on peak area) between samples and calibration points of two different transitions must not exceed 30%.

## Results and Discussion

### Occurrence of 1,4-Dioxane in Raw and Treated Water

Of the 587 water samples (raw and treated water) analysed for 1,4-dioxane, only 7% had concentrations above the LOQ (0.15 µg/L). In general, 1,4-dioxane was detected predominantly in additional samples potentially contaminated by 1,4-dioxane (63%), clearly indicating sites affected by industrial activities related to 1,4-dioxane. Greater flow samples (25%) and randomly selected samples from drinking water source samples (12%) were less affected.

**Fig. 2:**
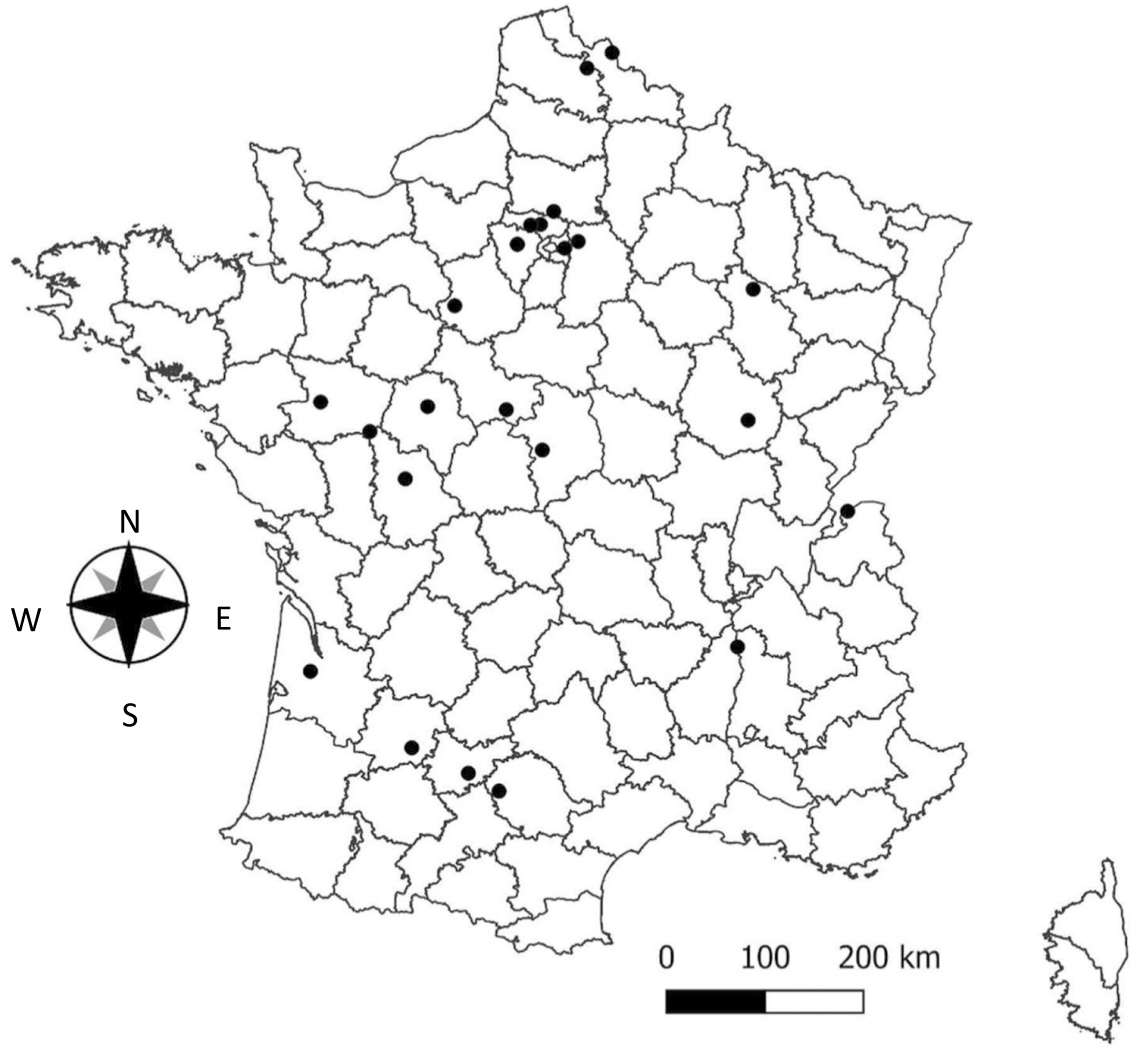
Map of sampling locations where 1,4-dioxane was quantified in treated water

Raw water for public water supplies includes both groundwater and surface water. In this study, due to the sampling strategy (Fig. 1), groundwater samples were predominant and accounted for 72% (214 sampling points) of the 300 raw water samples collected. 1,4-Dioxane was detected more often in surface water samples (9%) than in groundwater samples (7%). As shown in Fig. 1, 11 groundwater sampling points had concentrations of 1,4-dioxane between 0.35 and 3 µg/L compared with four surface water sampling points. The occurrence of 1,4-dioxane in raw (groundwater and surface water) and treated water was practically the same (Fig. 1). Concentrations of 1,4-dioxane ranging from 0.35 to 1 µg/L were predominant in nine and 11 sampling points of raw and treated water, respectively. Table 2 summarizes the positive results for 1,4-dioxane in raw and treated water obtained from each *département* during this sampling campaign. The sampling location where 1,4-dioxane was quantified in treated water is represented in Fig. 2.

**Table 2:** Summary of analytical results of 1,4-dioxane in raw and treated water samples collected during the sampling campaign from October 2020 to February 2022

Locations	Type of water	1,4-Dioxane (µg/L)
Site A1	GW	0.29
	TW	0.28
Site A2	SW	0.19
	TW	0.18
Site B1^a^	GW	0.30
	TW	2.46
Site C1^a^	GW	1.28
	TW	0.30
Site C2	GW	0.47
	TW	0.45
Site C3	GW	1.92
	TW	1.87
Site C4	SW	1.01
	TW	0.59
Site D1^a^	GW	0.29
	TW	< 0.15
Site D2	SW	0.32
	TW	0.15
Site D3	GW	0.47
	TW	0.46
Site E1	SW	1.01
	TW	0.68
Site E2^a^	SW	2.85
	TW	0.94
Site F1^a^	GW	0.79
	TW	< 0.15
Site F2	GW	0.43
	TW	0.35
Site F3^a^	GW	< 0.15
	TW	0.55
Site G1^a^	GW	0.26
	TW	< 0.15
Site G2	GW	0.40
	TW	0.42
Site G3	GW	0.48
	TW	0.51
Site H1^a^	GW	1.34
	TW	0.31
Site I2^a^	GW	1.20
	TW	1.39
Site I3^a^	SW	0.78
	TW	0.41
Site I4^a^	GW	0.20
	TW	0.18
Site I5	SW	0.20
	TW	< 0.15

*GW* groundwater, *SW* surface water; *TW* treated water

^a^Drinking water treatment plant supplied by more than one raw water source

With regard to the raw water samples, the groundwater resources contained concentrations of 1,4-dioxane ranging from 0.20 µg/L (site I4) to 1.92 µg/L (site C3). At site C3, the concentration of 1,4-dioxane was 5.5 times higher than the US EPA reference level (0.35 µg/L). Based on information provided by the Regional Health Agency (ARS—*Agence Régionale de Santé*), the groundwater of this site was polluted in the 1990s by industrial discharge containing chlorinated solvents such as 1,1,1-trichloroethane (TCA) and trichloroethylene (TCE). The industrial site was dedicated to the production of industrial fluids for the automobile industry. Although the industrial use of chlorinated solvents ceased in 2004, 1,4-dioxane is still present in groundwater resources. As previously reported by Adamson et al. (2015), 1,4-dioxane is a persistent compound in groundwater where chlorinated solvents are present and does not sorb strongly to aquifer solids. According to the ARS, historical use of chlorinated solvents was also identified at other sites investigated during this campaign, where 1,4-dioxane was systematically detected. Concentrations of 1,4-dioxane of 0.43, 1.20 and 1.28 µg/L were found in the groundwater resources of sites F2, I2 and C1, respectively. According to the ARS, the well at site C1 is located on a former industrial site. When the well was commissioned, the water contained traces of volatile organic compounds (VOCs). Site I2 was also heavily contaminated with chlorinated solvents, particularly TCE, by a pharmaceutical company in the 1980s. The soil was decontaminated in 1990, but 1,4-dioxane is still present in the groundwater. At site F2, bentazone and VOCs in groundwater have been monitored since 2010 by the ARS. There is an industrial site producing pesticides not too far from the water catchment. Previous studies have reported the cooccurrence of 1,4-dioxane with chlorinated volatile organic compounds (CVOCs) (ITRC 2021; Karges et al. 2018) and some have suggested that CVOCs inhibit the natural attenuation of 1,4-dioxane in groundwater (Adamson et al. 2015, 2022; Zhang et al. 2016). At site H1, groundwater had a 1,4-dioxane concentration of 1.34 µg/L. A company producing food contact materials and two facilities classified as environmentally regulated are located on the site, close to the water catchment area. These facilities are all industrial or agricultural activities subject to specific French regulations because they are likely to pollute the environment (water, soil, air).

It has been reported that groundwater is usually more contaminated than surface water (Adamson et al. 2017, 2021; Godri Pollitt et al. 2019; Karges et al. 2018). 1,4-Dioxane readily migrates to groundwater and matrix diffusion occurs in aquifers before other contaminants due to its high solubility in water and low log *K*_oc_ (USEPA 2014). However, the maximum concentration of 1,4-dioxane in surface water was observed at site E2 with 2.85 µg/L (Table 2). Several waste water treatment plants (WWTPs) are located upstream of the water intake of this river and in its tributaries. In Germany, elevated levels of 1,4-dioxane in surface waters have been attributed to municipal or industrial WWTPs (De Boer et al. 2022; Karges et al. 2022; Stepien et al. 2014). According to Dawson et al. (2022)) and Doherty et al. (2023)), consumers’ use of household and personal care products containing ethoxylated ingredients is a constant and significant source of 1,4-dioxane in surface water that is not removed by conventional wastewater treatments. At sites C4 and E1, both surface water samples revealed a 1,4-dioxane concentration of 1.01 µg/L. No information was obtained for either site, so the presence of 1,4-dioxane remains unexplained at this time. Field investigations need to be carried out to identify the sources of contamination.

Concerning treated water (Fig. 2), only three sites (B1, C3 and I2) had 1, 4-dioxane concentrations higher than 1 µg/L, with a maximum concentration of 2.46 µg/L at site B1 (Table 2). However, the associated raw water (groundwater) of site B1 showed a 1,4-dioxane concentration eight times lower. This was also the case for site I2, where the concentration of 1,4-dioxane was slightly higher in treated water (1.39 µg/L) than in groundwater (1.20 µg/L). At sites B1 and I2, 1,4-dioxane concentrations in treated water were higher than in raw water samples. This could be explained by the fact that the drinking water treatment plants (DWTPs) are supplied by more than one raw water source. Consequently, the most contaminated raw water source of these DWTPs may not have been analysed due to our initial sampling strategy. In order to confirm this hypothesis, complementary investigations were carried out on sites B1 and I2 in May 2022. Accordingly, all raw water sources supplying the DWTPs and their corresponding treated water were sampled. Moreover, at site I2, a DWTP not previously tested was also investigated in addition to the water samples initially analysed; this was called site I2a.

As shown in Table 3, the concentrations of 1,4-dioxane initially observed (Table 2) for raw water (GW1*) and treated water (TW*) at site B1 were confirmed to be of the same order of magnitude (0.24 and 2.01 µg/L). The additional raw water well of the DWTP (GW2) was found to be the most contaminated, with a 1,4-dioxane concentration of 3.5 µg/L. At site I2, the additional well analysed (GW2) was also the most polluted, with 1.42 µg/L of 1,4-dioxane. At the additional monitoring site, I2a, all groundwater samples were positive for 1,4-dioxane, with a maximum concentration of 4.8 µg/L. For site I2, as mentioned above, we learnt after analysis that these high levels were undoubtedly linked to pollution by chlorinated solvents in the 1980s. For sites B1 and I2a, the origin of the contamination has not yet been clearly identified.

**Table 3:** Concentrations of 1,4-dioxane found in raw and treated water at sites B1, I2 and I2a in May 2022

Locations	Type of water	1,4-Dioxane (µg/L)
Site B1	GW1*	0.24
	GW2	3.50
	TW*	2.01
Site I2	GW1*	1.26
	GW2	1.42
	TW*	1.43
Site I2a	GW1	4.80
	GW2	0.66
	GW3	0.48
	GW4	0.85
	GW5	1.02
	GW6	0.97
	GW7	0.26
	TW	3.16

*GW* groundwater, *TW* treated water

*Samples analysed in the initial sampling campaign from October 2020 to February 2022 (results in Table 2) and reanalysed in May 2022

### Efficiency of Drinking Water Treatment Plants

The preliminary evaluation of DWTP removal efficiency was only investigated at sites where treated water was produced from a single raw water source (Table 4) representing 54% of the 1,4-dioxane positive sites. At site C3, the concentrations of 1,4-dioxane in raw and treated water were equivalent (around 1.9 µg/L), showing that chlorination is not effective in removing 1,4-dioxane. This observation was confirmed at five other DWTPs (A1, C2, D3, G2 and G3). The levels of 1,4-dioxane were also practically unchanged after chloride disinfection of the water. As previously reported, the oxidation potential of hypochlorous acid is not strong enough to break down 1,4-dioxane and could potentially generate chlorinated 1,4-dioxane by-products (Deborde and von Gunten 2008; Kikani et al. 2022; NJDWQI 2022). Oxidants containing hydroxyl radicals, such as hydrogen peroxide, have been reported to be more effective for 1,4-dioxane degradation when combined with heat and/or UV irradiation and/or ozonation (Broughton et al. 2019; Godri Pollitt et al. 2019). However, the efficiency of these oxidation processes is highly dependent on several water quality parameters, such as natural organic matter, alkalinity, and chloramine levels (Masjoudi and Mohseni 2023). In addition, the oxidation reaction must be strictly controlled, as an incomplete reaction can lead to high levels of by-products (Broughton et al. 2019; NJDWQI 2022).

**Table 4:** Preliminary evaluation of removal efficiency levels at DWTPs on sampling sites from October 2020 to February 2022

Locations	Type of water	1,4-Dioxane (µg/L)	Treatment	Removal ratio (%)
Site A1	RW	0.29	C	− 2
	TW	0.28		
Site A2	RW	0.19	UFOACC	− 6
	TW	0.18		
Site C2	RW	0.47	C	− 5
	TW	0.45		
Site C3	RW	1.92	C	− 3
	TW	1.87		
Site C4	RW	1.01	SFOACC	− 41
	TW	0.60		
Site D2	RW	0.32	SFOACC	− 53
	TW	0.15		
Site D3	RW	0.47	C	− 1
	TW	0.46		
Site E1	RW	1.01	SFOACC	− 32
	TW	0.68		
Site F2	RW	0.43	ACC	− 19
	TW	0.35		
Site G2	RW	0.40	C	5
	TW	0.42		
Site G3	RW	0.48	C	8
	TW	0.51		

*RW* raw water, *TW* treated water, *C* chlorination, *UFOACC* ultrafiltration, ozonation, activated carbon and chlorination, *SFOACC* sand filtration, ozonation, activated carbon and chlorination, *ACC* activated carbon and chlorination

The use of activated carbon followed by chlorination (Site F2) showed little effectiveness in removing 1,4-dioxane concentration, as it led to a decrease of only 19%. Similar removal efficiency percentages (12–18%) using this treatment have been found previously (Carrera et al. 2019; Schoonenberg Kegel et al. 2010). Activated carbon filtration cannot be expected to effectively remove a significant amount of 1,4-dioxane due to its low adsorption potential and high miscibility with water (Stepien et al. 2014). Over time, 1,4-dioxane uptake is displaced by substances that have a greater affinity for the granular activated carbon (GAC). The breakthrough of 1,4-dioxane from the GAC may depend on the concentrations of other substances and background organic matter (Zietzschmann et al. 2016). A decrease of 32% in 1,4-dioxane concentration was observed at site E1, where raw water was treated by sand filtration, ozonation, activated carbon and chlorination. Carrera et al. (2019)) reported similar removal efficiency (34%) using this conventional treatment. Slightly higher decreases were observed at sites C4 and D2 (41 and 53%). These three sites (E1, C4 and D2) added a pre-ozonation step before sand filtration. The residual ozone levels after the water treatment were similar (0.3–0.4 mg/L). The ozone contact time could possibly explain the 10% variation in total removal efficiency between these three sites. However, the size range and age of the GAC filters could also affect removal efficiency, which is in competition with organic matter and other compounds. To know the GAC’s efficiency more precisely, it would be necessary to know when the GAC filters were regenerated and their iodine number, which indicates the pore volume available for adsorption. Virtually unchanged 1,4-dioxane concentrations were observed when ultrafiltration, ozonation, activated carbon and chlorination (site A2) were applied and for the moment, no explanation has been found. Further investigations are needed, and additional information on the DWTP at this site, such as GAC status, ozone dose and contact time, is required to explain the removal of 1,4-dioxane. As previously reported, our research confirms that conventional drinking water treatments, such as activated carbon adsorption, air stripping, membrane filtration and classical oxidation, have limited effectiveness in removing 1,4-dioxane (Carrera et al. 2019; Godri Pollitt et al. 2019). Advanced oxidation and biological treatments have been reported as promising approaches for the remediation of 1,4-dioxane from contaminated water (Kikani et al. 2022; McElroy et al. 2023; Tang, 2023).

## Conclusions

This study provides an overview of the occurrence of 1,4-dioxane in public water supplies in France. Concentrations of 1,4-dioxane were below the LOQ of the analytical method (0.15 µg/L) in more than 90% of the resources investigated. A few groundwater sites were contaminated with 1,4-dioxane, but those identified were associated with industrial sites. The maximum concentration of 1,4-dioxane observed in raw water was 4.8 µg/L, and the concentration in the associated treated water was 3.16 µg/L. The low efficiency of drinking water plants was shown in our preliminary evaluation, confirming that conventional water treatments do not completely remove 1,4-dioxane from water. These results are consistent with previous studies carried out in Germany and the USA. Further research is required to confirm the source of contamination, understand the transport and fate of 1,4-dioxane in water systems and more precisely evaluate the removal efficiency of 1,4-dioxane in drinking water treatment plants in order to estimate the French population’s exposure to 1,4-dioxane and establish a guideline value.

## Supplementary Information

Below is the link to the electronic supplementary material.

Supplementary file1 (DOCX 142 kb)
