# Supplementary material for: France-Wide Monitoring of 1,4-Dioxane in Raw and Treated Water: Occurrence and Exposure Via Drinking Water Consumption
Source: Arch Environ Contam Toxicol. 2024 Jul 31;87(2):95–104. doi: 10.1007/s00244-024-01078-6 (PMC11377507; doi:10.1007/s00244-024-01078-6)
Supplement: Supplementary file 1 — Supplementary file1 (DOCX 142 kb) [file 244_2024_1078_MOESM1_ESM.docx]

France-wide monitoring of 1,4-dioxane in raw and treated water: Occurrence and exposure via drinking water consumption

Cristina Bach^*^, Virginie Boiteux and Xavier Dauchy

ANSES, Nancy Laboratory for Hydrology, Water Chemistry Unit, 40 rue Lionnois, 54000 Nancy, France

*Corresponding author: [cristina.bach@anses.fr](mailto:cristina.bach@anses.fr)

**Table 1S**: Chemical properties of 1,4-dioxane (USEPA, 2014).

| **Parameter** | **Value** |
| --- | --- |
| *Chemical Formula* | C_4_H_8_O_2_ |
| *CAS Number* | 123-91-1 |
| *Molecular weight* | 88.11 g/mol |
| *Water solubility* | Miscible |
| *Octanol-water partition coefficient (log K_ow_)* | -0.27 |
| *Organic carbon partition coefficient (log K_oc_)* | 1.23 |

**Table 2S:** Non-comprehensive summary of extraction techniques for the determination of 1,4-dioxane in aqueous samples.

| **Extraction technique** | **Limit of quantification** | **References** |
| --- | --- | --- |
| Liquid-liquid extraction | From 1 to 3µg/L | USEPA (2006); USEPA (2018) |
| Solid-phase extraction (charcoal adsorbents) | From 0.034 to 0.5 µg/L | USEPA (2008); USEPA (2015); Carrera et al. (2017); Karges et al. (2020); Stepien and Püttmann (2013) |
| Purge & Trap | 0.15 µg/L | Sun et al. (2016) |
| Headspace | 0.07 µg/L | Hong et al. (2014) |
| SPME | From 0.5 to 1.2 µg/L | Nakamura and Daishima (2005); Shirey and Linton (2006) |


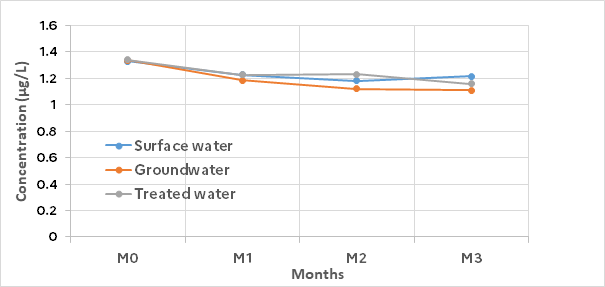
**Figure 1S:** Stability study of 1,4-dioxane at a concentration of 1µg/L in surface water, groundwater and treated water with sodium thiosulphate monitored at 4°C at M1 (1 month), M2 (2 months), and M3 (3 months).


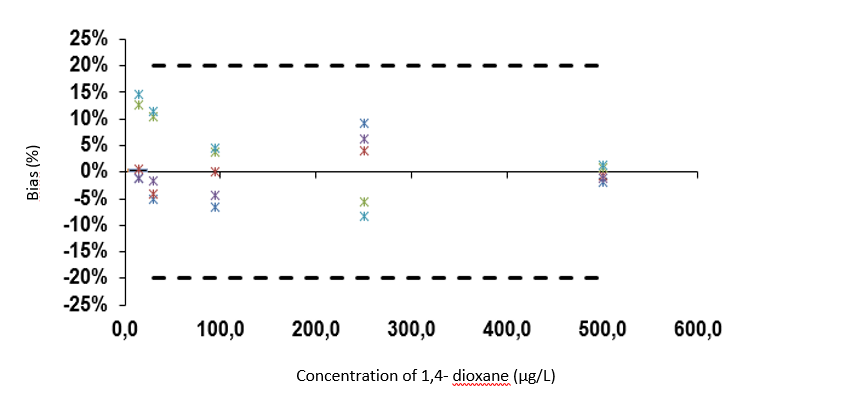
**Figure 2S**. Calculated bias between the experimental and the theoretical concentration of 1,4-dioxane for each calibration point.


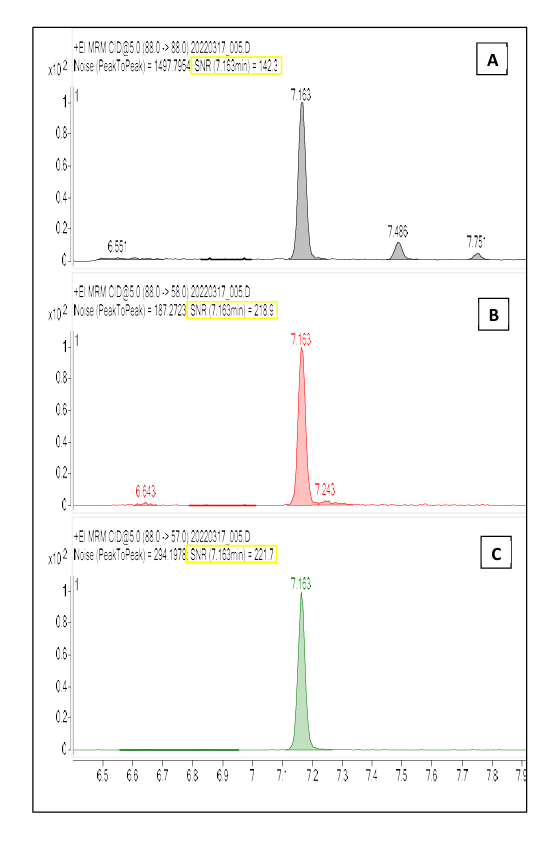


**Figure 3S:** GC-MS/MS chromatograms of the quantifier (A) and qualifier (B and C) MRM transitions monitored for 1,4-dioxane at 15 µg/L in DCM:EtAc (80:20)(v/v). This concentration corresponds to the LOQ (0.15 µg/L) after SPE extraction (concentration factor of 100). The signal-to-noise ratio (SNR) has been highlighted in each chromatogram.

**Table 3S:** Recovery study of 1,4-dioxane in three matrices (groundwater, surface water and drinking water).

| **Spiking concentration of 1,4-dioxane (µg/L)** | 0.15 | 0.75 | 4.00 |
| --- | --- | --- | --- |
| **Average (µg/L)** | 0.18 | 0.84 | 4.57 |
| **Relative bias (%)** | 17% | 13% | 14% |
| **Mean recovery (%)** | **117%** | **113%** | **114%** |

**Table 4S:** Recovery and standard deviation (%) for 1,4-dioxane according to the type of water during the sampling campaign from October 2020 to February 2022.

| **Type of water** | **Number of spiked samples at 1 µg/L** | **Recovery ± standard deviation (%)** |
| --- | --- | --- |
| Treated water | 47 | **115 ± 21** |
| Groundwater | 29 | **114 ± 18** |
| Surface water | 16 | **120 ± 11** |

Carrera G, Vegué L, Boleda MR, Ventura F (2017) Simultaneous determination of the potential carcinogen 1,4-dioxane and malodorous alkyl-1,3-dioxanes and alkyl-1,3-dioxolanes in environmental waters by solid-phase extraction and gas chromatography tandem mass spectrometry. J Chromatogr A 1487:1-13. https://doi.org/10.1016/j.chroma.2017.01.015.

Hong S-H, Lee J-B, Lee S-H, Hyun-Hee L, Shin H-S (2014) Measurement of 1,4-dioxane in surface water by headspace GC-MS. Anal Sci Technol 27:22-26. https://doi.org/10.5806/AST.2014.27.1.22.

Karges U, Ott D, De Boer S, Püttmann W (2020) 1, 4-Dioxane contamination of German drinking water obtained by managed aquifer recharge systems: Distribution and main influencing factors. Sci Total Environ 711:134783. https://doi.org/10.1016/j.scitotenv.2019.134783.

Nakamura S, Daishima S (2005) Simultaneous determination of 22 volatile organic compounds, methyl-tert-butyl ether, 1,4-dioxane, 2-methylisoborneol and geosmin in water by headspace solid phase microextraction-gas chromatography–mass spectrometry. Anal Chim Acta 548:79-85. https://doi.org/10.1016/j.aca.2005.05.077.

Shirey RE, Linton CM (2006) The extraction and analysis of 1,4-dioxane from water using solid-phase microextraction coupled with gas chromatography and gas chromatography-mass spectrometry. J Chromatogr Sci 44:444-450. https://doi.org/10.1093/chromsci/44.7.444.

Stepien DK, Püttmann W (2013) Simultaneous determination of six hydrophilic ethers at trace levels using coconut charcoal adsorbent and gas chromatography/mass spectrometry. Anal Bioanal Chem 405:1743-1751. https://doi.org/10.1007/s00216-012-6571-9.

Sun M, Lopez-Velandia C, Knappe DR (2016) Determination of 1, 4-dioxane in the Cape Fear River watershed by heated purge-and-trap preconcentration and gas chromatography–mass spectrometry. Environ Sci Technol 50:2246-2254. https://doi.org/10.1021/acs.est.5b05875.

USEPA (2006) Method 8260C: Volatile organic compounds by gas chromatography-mass spectrometry. U.S. Environmental Protection Agency. Revision 3.

USEPA (2008) Method 522: Determination of 1,4-dioxane in drinking water by solid phase extraction (SPE) and gas chromatography/mass spectrometry (GC/MS) with selected ion monitoring (SIM). U.S. Environmental Protection Agency.

USEPA (2014) EPA 505-F-14-011: Technical fact sheet - 1,4-Dioxane. Office of solid waste and emergency response. U.S. Environmental Protection Agency.

USEPA (2015) Method 541: Determination of 1-butanol, 1,4-dioxane, 2-methoxyethanol and 2-propen-1-ol in drinking water by solid phase extraction and gas chromatography-mass spectrometry. U.S. Environmental Protection Agency.

USEPA (2018) Method 8270E: Semivolatile organic compounds by gas chromatography-mass spectrometry. U.S. Environmental Protection Agency.
